# Association between nasal and nasopharyngeal bacterial colonization in early life and eczema phenotypes

**DOI:** 10.1111/cea.13869

**Published:** 2021-04-06

**Authors:** Chen Hu, Liesbeth Duijts, Evelien R. van Meel, Kirsten I. M. Looman, Jessica C. Kiefte‐de Jong, Luba M. Pardo, DirkJan Hijnen, Suzanne G. M. A. Pasmans, Johan C. de Jongste, Henriette A. Moll, Tamar Nijsten

**Affiliations:** ^1^ The Generation R Study Group University Medical Center Rotterdam Erasmus MC Rotterdam The Netherlands; ^2^ Department of Dermatology University Medical Center Rotterdam Erasmus MC Rotterdam The Netherlands; ^3^ Division of Respiratory Medicine and Allergology Department of Pediatrics University Medical Center Rotterdam Erasmus MC Rotterdam The Netherlands; ^4^ Division of Neonatology Department of Pediatrics University Medical Center Rotterdam Erasmus MC Rotterdam The Netherlands; ^5^ Department of Pediatrics University Medical Center Rotterdam Erasmus MC Rotterdam The Netherlands; ^6^ Department of Epidemiology University Medical Center Rotterdam Erasmus MC Rotterdam The Netherlands; ^7^ Department of Public Health and Primary Care Leiden University Medical Center Leiden The Netherlands

**Keywords:** atopic dermatitis, birth cohort, nasal bacteria, *S. aureus* colonization

## Abstract

**Background:**

An association has been reported between early life *Staphylococcus aureus* nasal carriage and higher risk of childhood eczema, but it is unclear whether this relationship is causal and associations with other bacterial species are unclear.

**Objective:**

To examine the associations of early life nasal and nasopharyngeal bacterial carriage with eczema phenotypes, and the direction of any associations identified.

**Methods:**

Among 996 subjects of a population‐based prospective cohort study, nasal swabs for *Staphylococcus  aureus*, and nasopharyngeal swabs for *Streptococcus pneumoniae*, *Moraxella catarrhalis* and *Haemophilus influenzae* were collected and cultured from age 6 weeks to 6 years. Never, early, mid‐, late transient and persistent eczema phenotypes were identified from parental‐reported physician‐diagnosed eczema from age 6 months until 10 years. Multinomial regression models and cross‐lagged models were applied.

**Results:**

*Staphylococcus aureus* nasal carriage at 6 months was associated with an increased risk of early transient and persistent eczema (OR (95% CI): 2.69 (1.34, 5.39) and 4.17 (1.12, 15.51)). The associations between *Staphylococcus aureus* nasal carriage and eczema were mostly cross‐sectional, and not longitudinal. No associations of *Staphylococcus pneumoniae*, *Moraxella catarrhalis* and *Haemophilus influenza* nasopharyngeal bacterial carriage with eczema and eczema phenotypes were observed (OR range (95% CI): 0.71 (0.35, 1.44) to 1.77 (0.84, 3.73)).

**Conclusions:**

Early life *Staphylococcus aureus* nasal carriage, but not *Staphylococcus pneumoniae*, *Moraxella catarrhalis* and *Haemophilus influenza* nasopharyngeal carriage, was associated with early transient and persistent eczema. *Staphylococcus aureus* nasal carriage and eczema were mostly cross‐sectionally associated, and not longitudinally, making a causal relationship in either direction unlikely.

## Graphical abstract

Early life *Staphylococcus aureus* nasal carriage was associated with an increased risk of early transient and persistent eczema. *S. aureus* nasal carriage and eczema were mostly cross‐sectionally associated, and not longitudinally, making a causal relationship in either direction unlikely. No associations of *Streptococcus pneumoniae*, *Moraxella catarrhalis* and *Haemophilus influenza* nasopharyngeal bacterial carriage with eczema and eczema phenotypes were observed.

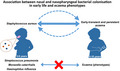

## 1 INTRODUCTION

Childhood eczema is a common chronic skin disorder with variable age of onset and persistence.^1^ We previously identified eczema phenotypes taking into account the variability of eczema onset and persistence within and between individuals over time.^2^ The use of eczema phenotypes, instead of the simplified dichotomous outcome of eczema, might better reflect the natural course of eczema and help understand their specific underlying risk factors. Both genetic and environmental factors seem to influence the development and persistency of eczema.^3^ Additionally, bacterial carriage of the main commensals *Staphylococcus aureus*, *Haemophilus influenzae*, *Moraxella catarrhalis* and *Streptococcus pneumoniae* in the nasal cavity and nasopharynx was suggested to be associated with eczema.^4^, ^5^ The nasal and nasopharyngeal area may function as important reservoirs for bacteria to spread to different body sites. In addition, competitive and cooperative inter‐bacterial, and host‐bacterial interactions affect the microbial colonization dynamics and the priming of the host's immune responses, and thereby altering the susceptibility of developing atopic diseases.^6^ A previous meta‐analysis of mainly hospital‐based cohorts showed that nasal carriage of *S. aureus* was associated with an increased risk of eczema in children and adults.^4^ We previously showed in a population‐based cohort that early life nasal carriage of *S. aureus* was associated with increased risk of eczema and eczema severity in children aged 1–2 years, but persistent effects at older ages were not clear.^7^ Also *Haemophilus influenzae*, *Moraxella* and *Streptococcus pneumoniae* in the nasopharynx are suggested to be associated with increased risk of eczema.^5^, ^8^, ^9^ However, studies only used vaccinations against *Haemophilus influenzae* and *Streptococcus pneumoniae*, not bacterial carriage, and were performed in hospital‐based or adult populations.^5^, ^8^, ^9^ Furthermore, it remains unclear whether bacterial nasal and/or nasopharyngeal carriage leads to increased risk of the development of eczema, is a consequence of eczema, or occurs simultaneously with eczema due to other mechanisms.^10^ Therefore, we aimed to examine the associations of early life bacterial nasal and nasopharyngeal carriage with eczema phenotypes from birth until age 10 years among 996 subjects of a population‐based prospective cohort study. Next, we aimed to disentangle whether the direction of associations was from bacterial nasal and nasopharyngeal carriage leading to an increased risk of eczema or reversely.

## 2 METHODS

### 2.1 Design

**FIGURE 1:**
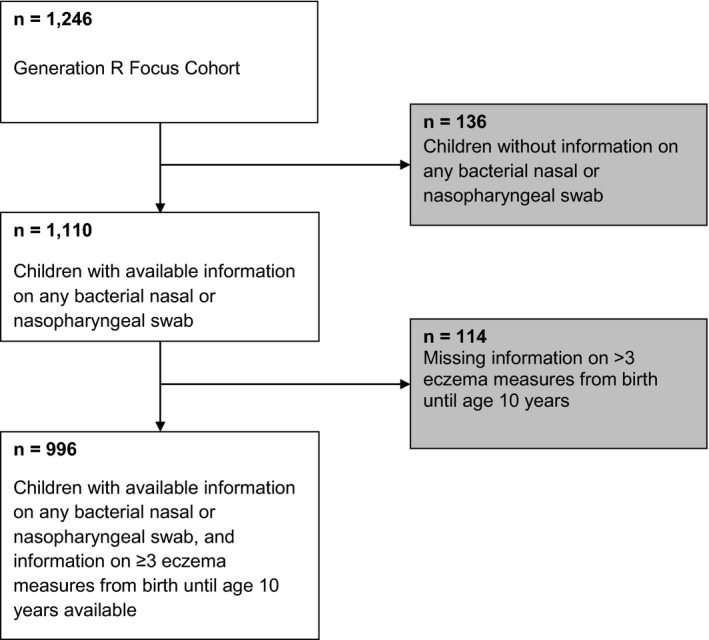
Flow chart of participants included for analysis

This study was embedded in the Generation R Study, a population‐based prospective cohort study from early foetal life onwards in Rotterdam, the Netherlands.^11^, ^12^ The study has been approved by the Medical Ethical Committee of the Erasmus MC, University Medical Centre in Rotterdam (MEC 198.782/2001/31; MEC 217.595/2002/202; MEC‐2007‐413; and MEC‐2012‐165). Written informed consent was obtained from all participants. A total of 996 children were included for the current analysis (Figure 1).

### 2.2 Bacterial nasal and nasopharyngeal carriage

Swabs of the nose and nasopharynx area were taken by trained research nurses at the research centre at ages 6 weeks, 6 and 14 months, and 2, 3 and 6 years, as previously described.^13^, ^14^ For this, sterile transport swabs with liquid Amies medium were used. Nasal swabs were put in phenol red mannitol broth at 35°C for 5 days. Material from tubes that turned yellow was plated on a blood agar plate with 5% sheep blood at 35°C for 1 day to isolate *Staphylococcus aureus*. Nasopharyngeal swabs were plated on a *Haemophilus* selective agar plate, a blood agar plate with 5% sheep blood and a chocolate agar plate for *Haemophilus influenzae*, *Moraxella catarrhalis* and *Streptococcus pneumoniae*, respectively. The plates were kept at 35°C in a CO_2_ rich environment for 2 days and assessed daily for growth of bacteria. Swabs were classified as either negative or positive for *S. aureus*, *H. influenzae*, *M. catarrhalis* or *S. pneumoniae*. Nasopharyngeal carriage with any bacteria was classified as positive when one of the bacteria *H. influenzae*, *M. catarrhalis* or *S. pneumoniae* was positive, and negative if all three were negative. Additionally, to study the bacterial nasopharyngeal carriage in detail, sensitivity analysis was performed on each of the three bacteria separately.

### 2.3 Eczema phenotypes

Information on physician‐diagnosed eczema was obtained from parental‐reported questionnaires at the ages of 6 months, and 1, 2, 3, 4 and 10 years (‘Was your child diagnosed with eczema in the last 6 months/last year by a general practitioner or physician in the hospital?’) (no; yes). In children with available data on physician‐diagnosed eczema on at least 3 time points, we previously identified five eczema phenotypes (never, early transient, mid‐transient, late transient and persistent eczema) using latent class growth analysis, in which missing data are handled by maximum likelihood algorithm.^2^ Subjects were assigned to the eczema phenotype for which they had the highest posterior probability. Children with early‐, mid‐ and late transient eczema had a high probability of developing eczema at approximately the age of 6 months, 2 years and 5 years, respectively, after which the eczema gradually declines. Children with persistent eczema had a high probability of eczema from birth until the age of 10 years. Ever eczema included those with early transient, mid‐transient, late transient or persistent eczema. Due to relative low number of subjects in the persistent eczema phenotype in the five eczema phenotypes model, we performed sensitivity analyses with stronger statistical stability using three eczema phenotypes (never, early‐mid transient and late‐persistent phenotype).

### 2.4 Covariates

Information on pet keeping and maternal psychiatric symptoms using the Global Severity Index (GSI) was obtained by questionnaires during pregnancy.^15^ The mode of delivery was obtained from midwives and hospital records. Postnatal questionnaires provided information on daycare attendance and antibiotic use in the first year after birth.

### 2.5 Statistical analysis

We compared characteristics of those included and not included in our study using Pearson's Chi‐square and Mann‐Whitney U tests. First, we examined the associations of bacterial nasal and nasopharyngeal carriage with ever eczema and with five eczema phenotypes from birth until age 10 years using logistic and weighted multinomial regression models, respectively. Weights were based on class probabilities. Next, cross‐lagged models were used to examine bidirectional associations of bacterial nasal carriage with eczema from birth until 10 years. Cross‐lagged models allow associations between two repeatedly measured variables to be examined in both directions simultaneously while accounting for continuity between the repeated measures over time. A conceptual model of the studied cross‐lagged associations is presented in Figure S1. For example, the effect estimates of the association of bacterial carriage at earlier age with eczema at later age will be adjusted for all earlier associations between and within bacterial carriages and eczema. With this method, we aimed to disentangle the predominant direction of the observed association between bacterial nasal carriage and eczema. We examined cross‐lagged effects, cross‐sectional effects and stability effects in the period from birth until age 3 years, and only cross‐lagged and stability effect in the period from 4 until 10 years due to the uneven distribution of repeated measures of bacterial nasal carriage and eczema at those ages. As a sensitivity analyses for increased statistical power, we applied generalized estimating equation (GEE) models with an unstructured and autoregressive correlation matrix to examine the associations between bacterial nasal and nasopharyngeal carriage at age 6 weeks with repeated measures of eczema from 6 months until 10 years. All analyses were adjusted for potential confounders, which were first selected from literature including known potential underlying biological mechanisms.^2^, ^16^, ^17^, ^18^ Next, confounders were selected if they were associated with both the exposure (bacterial nasal/nasopharyngeal carriage) and the outcome (eczema phenotypes), and were not within the causal pathway based on epidemiological concept. Additionally, they were included if they changed the effect estimates or the unadjusted analyses with ≥10% in adjusted analyses. Family history of atopic diseases, maternal age, parity and education, and child's gestational age, birth weight, sex and breastfeeding did not meet our defined statistical criteria of confounding, and therefore, were not included in the models. For better interpretation, we adjusted all analyses models for the same confounders. We assumed that data were missing at random. Twenty data sets were created to handle missing data in covariates (≤12%) using multiple imputation by chained equations. Missing data in bacterial nasal and nasopharyngeal carriage and eczema were not imputed. The size and direction of the effect estimates were similar when we used complete‐case analyses, and therefore, we only present the results based on imputed data. We did not adjust for multiple testing in the main analyses (nasal carriage with *S. aureus* and nasopharyngeal carriage with any bacteria), because the bacterial carriages were examined under the same hypothesis. For the sensitivity analysis of the separate nasopharyngeal bacteria, we corrected for multiple testing using alpha .05 divided by the effective independent number of tests calculated based on the correlation structure between the bacteria.^19^ All measures of association are presented as odds ratios (OR) together with their corresponding 95% confidence intervals (95%CI). Imputation and regression analyses were performed using the packages ‘mice’ (version 3.6.0), ‘stats’ (version 3.6.1) and ‘nnet’ (version 7.3.12), cross‐lagged analyses were performed in Mplus (version 8.2), and using package ‘MplusAutomation’ (version 0.7‐3), and GEE analyses were performed using the package ‘geepack’ (version 1.2‐1) in R version 3.6.1.^20^, ^21^, ^22^, ^23^, ^24^, ^25^

## 3 RESULTS

### 3.1 Subject characteristics

Characteristics of children and their mothers are shown in Table 1. Compared with children included in the analysis, those not included had mothers who had more psychiatric symptoms during pregnancy (Table S1). The number of children eligible for inclusion during follow‐up was 1190 children at ages 6 weeks to 4 years, 1166 children at age 5 years, and 1109 at age 10 years. Physician‐diagnosed eczema ranged from 13.4% at age 6 months to 6.0% at age 10 years (Table S1).

**TABLE 1:** Characteristics of children and their mothers (*n *= 996)

	All (*n* = 996)	Never eczema (*n* = 768)	Ever eczema (*n* = 228)
Maternal characteristics			
Pet keeping, yes % (*n*)	43.1 (429)	42.4 (326)	44.3 (101)
Pyschiatric symptoms, median (IQR)	0.12 (0.06, 0.23)	0.10 (0.06, 0.21)	0.13 (0.04, 0.25)
Mode of delivery % (*n*)			
Vaginal	85.1 (848)	86.4(664)	80.9 (184)
Primary caesarian section	6.6 (66)	6.0 (46)	8.3 (19)
Secondary caesarian section	8.2 (82)	7.6 (58)	10.9 (25)
Children characteristics			
Daycare attendance, yes % (*n*)	68.5 (682)	68.8 (525)	67.1 (153)
Antibiotic use, yes % (*n*)	35.7 (356)	34.7 (266)	38.6 (88)
*S*. *aureus* carriage, yes % (*n*/total *n*)^a^			
Age 6 weeks	52.7 (302/573)	51.9 (232)	55.6 (70)
Age 6 months	20.9 (150/718)	18.2 (101)	30.2 (49)
Age 1 year	14.5 (98/676)	14.9 (78)	13.0 (20)
Age 2 years	13.4 (79/590)	12.1 (54)	17.4 (25)
Age 3 years	14.7 (89/604)	13.5 (62)	18.5 (27)
Age 6 years	28.0 (238/850)	26.3 (173)	34.0 (65)
Nasopharyngeal carriage with any bacteria, yes % (*n*/total *n*)^a^			
Age 6 weeks	22.9 (131/573)	21.9 (98)	26.2 (33)
Age 6 months	61.1 (438/717)	60.8 (338)	62.1 (100)
Age 1 year	67.0 (453/676)	67.4 (352)	65.6 (101)
Age 2 years	63.6 (375/590)	63.5 (283)	63.9 (92)
Age 3 years	50.0 (302/604)	50.0 (229)	50.0 (73)
Age 6 years	36.9 (314/850)	36.6 (241)	38.2 (73)
Eczema phenotypes, % (*n*)*			
Never eczema	77.1 (768)	100.0 (768)	0.0 (0)
Early transient eczema	7.5 (75)	0.0 (0)	32.9 (75)
Mid‐transient eczema	6.7 (67)	0.0 (0)	29.4 (67)
Late transient eczema	7.1 (71)	0.0 (0)	31.1 (71)
Persistent eczema	1.5 (15)	0.0 (0)	6.6 (15)

Note

Values are means (SD), valid percentages (absolute numbers) or medians (95% range) based on imputed data.

^a^ Nasal carriage of *S. aureus* and nasopharyngeal carriage with any bacteria (*H. influenzae*, *M. catarrhalis* or *S. pneumoniae*) were not imputed and were missing (%) for the following ages: 43% at 6 weeks, 28% at 6 months, 32% at 1 year, 41% at 2 years, 39% at 3 years and 15% at 6 years. Eczema phenotypes had no missing values.

### 3.2 Early life bacterial nasal and nasopharyngeal carriage and eczema phenotypes

Compared with never eczema and no nasal carriage of *S. aureus*, nasal carriage of *S. aureus* at age 6 months was associated with an increased risk of ever eczema (OR (95% CI): 2.01 (1.33, 3.02); Table 2). Nasal carriage of *S. aureus* at other ages, and nasopharyngeal carriage with any bacteria was not associated with ever eczema. When examining eczema phenotypes, nasal carriage of *S. aureus* at age 6 months was associated with an increased risk of early transient and persistent eczema, compared with no nasal carriage of *S. aureus* and never eczema phenotype (OR (95% CI): 2.69 (1.34, 5.39) and 4.17 (1.12, 15.51), respectively; Table 2). Nasal carriage of *S. aureus* at ages 6 weeks, and 1, 2, 3, and 6 years was not associated with eczema phenotypes. Similar size and direction of estimates were observed when using the regrouped three eczema phenotypes, although the associations of nasal carriage of *S. aureus* at age 6 months with late‐persistent eczema attenuated into non‐significant (Table S2). We found no associations of nasopharyngeal carriage with any bacteria between the ages of 6 weeks and 6 years with eczema phenotypes (Table 2). When we studied the nasopharyngeal carriage with *H. influenzae*, *M. catarrhalis* and *S. pneumoniae* separately in a sensitivity analyses, only nasopharyngeal carriage of *H. influenzae* at age 6 months was associated with an increased risk of early transient eczema phenotype (2.09 (1.03, 4.24); Table S3). This association attenuated to non‐significant after correcting for multiple testing. We observed no associations of nasal carriage of *S*. *aureus* or nasopharyngeal carriage with any bacteria at ages 6 weeks with overall eczema from birth until age 10 years in the sensitivity analyses with GEE models (data not shown).

**TABLE 2:** Associations of bacterial nasal and nasopharyngeal carriage with ever eczema and eczema phenotypes

	Ever eczema	Never eczema	Early transient eczema	Mid‐transient eczema	Late transient eczema	Persistent eczema
	Odds ratio (95% Confidence Interval) (*n *= 228)	Odds ratio (95% Confidence Interval) (*n *= 768)	Odds ratio (95% Confidence Interval) (*n *= 75)	Odds ratio (95% Confidence Interval) (*n *= 67)	Odds ratio (95% Confidence Interval) (*n *= 71)	Odds ratio (95% Confidence Interval) (*n *= 15)
*S*. *aureus* carriage						
Age 6 weeks	1.21 (0.80,1.84)	Reference	1.42 (0.66, 3.09)	1.59 (0.57, 4.46)	0.93 (0.47, 1.85)	0.82 (0.16, 4.04)
Age 6 months	2.01 (1.33, 3.02)**	Reference	2.69 (1.34, 5.39)**	1.90 (0.74, 4.91)	1.48 (0.69, 3.21)	4.17 (1.12, 15.51)*
Age 1 year	0.88 (0.51, 1.52)	Reference	0.82 (0.30, 2.24)	0.97 (0.28, 3.42)	0.69 (0.23, 2.07)	1.14 (0.21, 6.38)
Age 2 years	1.64 (0.96, 2.77)	Reference	1.29 (0.50, 3.37)	2.02 (0.67, 6.06)	1.20 (0.43, 3.35)	2.08 (0.30, 14.15)
Age 3 years	1.57 (0.94, 2.61)	Reference	1.68 (0.66, 4.24)	1.64 (0.53, 5.07)	1.20 (0.48, 2.99)	2.25 (0.35, 14.60)
Age 6 years	1.40 (0.98, 2.00)	Reference	1.33 (0.68, 2.6)	1.22 (0.54, 2.78)	1.74 (0.94, 3.21)	1.82 (0.50, 6.63)
Nasopharyngeal carriage with any bacteria^a^						
Age 6 weeks	1.36 (0.85, 2.20)	Reference	1.01 (0.40, 2.60)	1.21 (0.38, 3.80)	1.77 (0.84, 3.73)	1.39 (0.23, 8.61)
Age 6 months	1.06 (0.72, 1.56)	Reference	1.11 (0.54, 2.27)	1.16 (0.45, 2.98)	0.80 (0.40, 1.61)	1.82 (0.45, 7.39)
Age 1 year	0.90 (0.59, 1.37)	Reference	1.12 (0.50, 2.48)	0.62 (0.24, 1.66)	0.98 (0.44, 2.17)	0.83 (0.19, 3.61)
Age 2 years	1.06 (0.70, 1.61)	Reference	0.71 (0.35, 1.44)	1.35 (0.50, 3.66)	1.05 (0.48, 2.29)	1.23 (0.22, 6.73)
Age 3 years	0.95 (0.64, 1.40)	Reference	1.53 (0.72, 3.22)	0.73 (0.29, 1.82)	1.02 (0.52, 1.97)	0.82 (0.17, 3.99)
Age 6 years	1.10 (0.79, 1.54)	Reference	1.06 (0.56, 2.00)	1.10 (0.51, 2.36)	1.23 (0.68, 2.23)	0.85 (0.23, 3.16)

Note

Values are odds ratios (OR) with 95% confidence interval from logistic and multinomial regression models on imputed data.

^a^ Nasopharyngeal bacteria include *H. influenzae*, *M. catarrhalis* or *S. pneumoniae*. Models were adjusted for maternal psychiatric symptoms, pet keeping, mode of delivery, daycare attendance and antibiotic use.

* *p*‐Value < .05.

** *p*‐Value < .01.

### 3.3 Direction of associations between bacterial nasal and nasopharyngeal carriage and eczema

**FIGURE 2:**
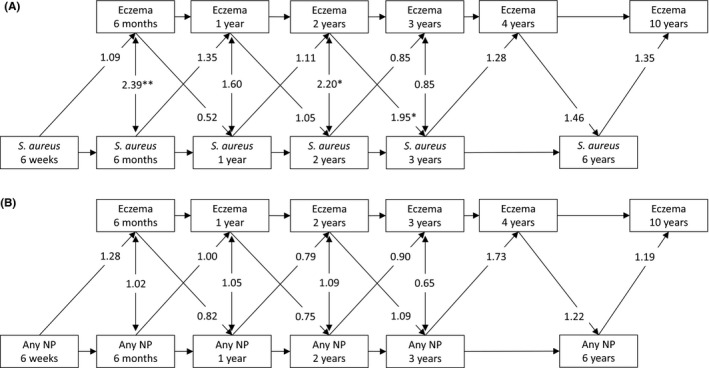
Bidirectional associations between nasal (A) *S. aureus* carriage and eczema, and (B) nasopharyngeal carriage with any bacteria (any NP), including *H. influenzae*, *M. catarrhalis* or *S. pneumoniae*, and eczema from birth until 10 years. Values are odds ratios (95% confidence interval) derived from logistic regression models, using cross‐lagged modelling. Models were adjusted for maternal psychiatric symptoms, pet keeping, mode of delivery, daycare attendance and antibiotic use. *p‐value < .05, **p‐value < .01. The corresponding 95% confidence intervals of the cross‐lagged and cross‐sectional effects, and the effect estimates of the stability effects are shown in Table S3

Figure 2 and Table S4 show the bidirectional associations between bacterial nasal and nasopharyngeal carriage and eczema as dichotomous outcome from age 6 weeks to 10 years using cross‐lagged models. Stability effect analysis showed that nasal carriage of *S. aureus* at earlier age was associated with increased risk of nasal carriage of *S. aureus* at later age between ages 6 weeks and 6 months, 2 to 3 years, and 3 to 6 years (OR (95% CI): 2.23 (1.36, 3.60), 2.39 (1.27, 4.48) and 1.73 (1.04, 2.89), respectively; Table S4). Stability effect analysis showed that eczema at earlier age was associated with eczema at later age between age 6 months and 10 years (OR (95% CI) range: 4.53 (1.99, 10.28) to 11.02 (6.36, 18.92)). Cross‐sectional effect analysis showed that nasal carriage of *S. aureus* was associated with eczema at ages 6 months and 2 years (OR (95% CI): 2.39 (1.38, 4.14) and 2.20 (1.16, 4.18), respectively; Figure 2A; Table S4). Cross‐lagged effect analysis showed that children with eczema at age 2 years had an increased risk of nasal carriage of *S*. *aureus* at age 3 years (1.95 (1.02, 3.71)), but not at other ages. Reversely, no associations were observed of nasal carriage of *S. aureus* with eczema. For nasopharyngeal carriage with any bacteria, stability effect analysis showed that nasopharyngeal carriage with any bacteria at earlier age was largely associated with increased risk of nasopharyngeal carriage with any bacteria at later age between age 6 weeks and 6 years (OR (95% CI) range: 1.28 (0.86, 1.92) to 3.32 (2.29, 4.81); Table S4). No cross‐sectional or cross‐lagged associations were observed between nasopharyngeal carriage with any bacteria and eczema (Figure 2B). Results from cross‐lagged models were similar in effect size and direction when examining nasopharyngeal carriage with *H. influenzae*, *M. catarrhalis* and *S. pneumoniae* separately (Table S5).

## 4 DISCUSSION

In this population‐based prospective cohort study, we observed that only nasal carriage of *S. aureus* at age 6 months was associated with an increased risk of ever eczema, and specifically with an increased risk of early transient and persistent eczema phenotypes until age 10 years. The direction of effects between nasal carriage of *S. aureus* and eczema was largely cross‐sectional, making causality either way unlikely. Nasopharyngeal bacterial carriage with *H. influenzae*, *M. catarrhalis* and/or *S. pneumoniae* from age 6 weeks until age 6 years was not associated with ever eczema or eczema phenotypes from birth until age 10 years.

### 4.1 Comparison with previous studies

A previous meta‐analysis, cohort and case‐control studies showed that nasal carriage of *S. aureus* was associated with an up to fivefold increased risk of eczema in children and adults.^4^, ^26^ We observed in our current study that nasal carriage of *S. aureus* at age 6 months was associated with an increased risk of ever eczema at age 10 years, which is in line with results of our previous study in children until age 2 years.^7^ We now additionally explored eczema phenotypes across childhood, taking the onset and persistence of eczema into account, and observed that nasal carriage of *S. aureus* at age 6 months was associated with an increased risk of early transient and persistent eczema. Nasal carriage of *S. aureus* at other ages until 6 years was not associated with ever eczema, and eczema phenotypes until age 10 years. Studies examining the direction of association between nasal carriage of *S. aureus* and eczema are scarce. Only two previous longitudinal studies, using one‐directional statistical methods, examined the association between skin carriage of *S. aureus* and eczema in children, and showed conflicting results.^27^, ^28^ Skin carriage of *S. aureus* 2 months prior to eczema onset was associated with an increased risk of eczema in a cohort of 149 children until age 2 years, while skin carriage of *S. aureus* at age 2 months was associated with a decreased risk of eczema in a nested case‐control study of 20 children.^27^, ^28^ The use of cross‐lagged models allowed us to examine the effects between nasal carriage of *S. aureus* and eczema in both directions (bidirectional), and we observed that this association was largely cross‐sectional, and not longitudinal.

Airway carriage of *H. influenzae*, *M. catarrhalis* and *S. pneumoniae* is suspected to play a role in the development of childhood atopic respiratory diseases by influencing the microbiota composition dynamics and/or the priming of the host's immune responses.^6^, ^16^, ^29^ Therefore, we hypothesized that early life nasal and/or nasopharyngeal carriage with *H. influenzae*, *M. catarrhalis* or *S. pneumoniae* might also be associated with eczema, however, few studies examined these associations. A cross‐sectional cohort study in children with eczema showed that nasal carriage of different *Moraxella* species was associated with both increased, and decreased risk of eczema severity.^5^ A previous case‐control and cohort study showed that adults with eczema had higher risks of severe *S. pneumoniae* infections, and children with eczema had a delayed response to *Pneumococcal* vaccine, respectively.^9^, ^30^ One cohort study showed that *H. influenzae* vaccination at age 6 months was associated with an increased risk of eczema at age 18 months.^8^ We observed no associations of nasopharyngeal carriage of *H. influenzae*, *M. catarrhalis* and *S. pneumoniae* with ever eczema, and eczema phenotypes on a population‐level. The difference between our results and the suggested positive associations of these specific bacteria with eczema in previous studies might be explained by differences in study population (general versus hospital‐based) and methods (microbial culture versus more sensitive rRNA sequencing and nasal carriage versus vaccination responses), and potential publication bias towards positive findings.

### 4.2 Interpretation of results

Children with early transient and persistent eczema have a high probability of eczema at approximately 6 months of age.^2^ Our observations of nasal carriage of *S. aureus* at age 6 months being associated with increased risks of early transient and persistent eczema, and not with mid‐transient or late transient eczema, suggest that nasal carriage of *S. aureus* is associated with active eczema, and not with eczema developing in later life. This hypothesis is also supported by the results from our cross‐lagged analyses that showed predominantly cross‐sectional associations between nasal carriage of *S. aureus* and eczema rather than causal associations from nasal carriage of *S. aureus* to eczema or vice versa. Previous experimental and case‐control studies showed that *S. aureus* might play a role in the development and worsening of eczema via the production of proteins, proteases and superantigens that can induce chronic inflammation of the skin.^31^, ^32^, ^33^ However, a recent review found insufficient evidence for beneficial effects of *S. aureus* reducing interventions on eczema.^10^ Therefore, *S. aureus* carriage or overgrowth might also be the consequence of a favourable skin environment in children with eczema. For instance, in eczema the skin has a higher pH compared to normal skin, and changes in skin microbiome composition may lead to more *S. aureus* growth.^31^ Another possibility is that underlying immune dysregulation or skin characteristics, such as dry skin due to reduced levels of natural moisturizing factor and loss of function mutation in the filaggrin gene, prior to eczema development might benefit *S. aureus* growth.^31^, ^34^, ^35^ Interestingly, nasal carriage of *S. aureus* at age 6 months, and not at older ages, was associated with eczema phenotypes. The prevalence of nasal carriage of *S. aureus* is highest at age 6 weeks and declines at age 6 months in children with and without eczema. However, the prevalence of nasal carriage of *S. aureus* is higher in children with eczema than in those without eczema at age 6 months. A possible explanation is that the maturation of the skin and immune system is disrupted and/or delayed especially in children with early‐onset eczema, which increases the susceptibility of *S. aureus* (over)growth on the nose, and perhaps also on other body sites.^31^ Future studies should focus on the microbiota on multiple body sites, such as nose, skin and gut in a longitudinal way, to further assess the relationship between bacteria and eczema.

### 4.3 Strengths and limitations

The strengths of this study include that it is embedded in a population‐based prospective designs with detailed and repeated information on bacterial nasal carriage and eczema, the use of eczema phenotypes and cross‐lagged models to statistically examine longitudinal associations. However, methodological limitations of this study must also be taken into account when interpreting the results. Selection bias might occur when the associations between bacterial nasal carriage and eczema phenotypes were different in children included versus those not included in the analysis. Second, we used nasal swabs to examine *S. aureus* carriage. While the nose is the most frequent carriage site, other *S. aureus* prevalent body sites, such as the axilla and perineum, were not examined, thereby limiting the generalizability to nasal carriage of *S. aureus* only.^36^, ^37^ Third, a relatively small number of children had nasal swabs, and not all of these had nasal swabs at every visit. Therefore, we were not able to cluster trajectories of bacterial nasal carriage in order to study the associations of bacterial nasal carriage phenotypes with eczema phenotypes. Fourth, non‐differential misclassification of eczema remains possible due to use of self‐reported questionnaires. Although these questionnaires have been validated for defining eczema in epidemiological research, cases with very mild eczema might be lacking since they are less likely to visit a physician.^38^ In addition, most children in population‐based settings have relatively mild disease, making it difficult to generalize findings to patients with moderate to severe eczema. Last, residual confounding might be present due to influencing factors not measured in our study. For example, the abundance of *S. aureus* and other nasopharyngeal bacteria, and horizontal transmission by surroundings could not be determined in our study.^36^ It might be that children with early transient and persistent eczema have skin conditions (i.e., pH level, natural moisturizing factor and filaggrin gene mutations) more suitable for *S. aureus* overgrowth, increased contact with parents due to frequent comforting, *S. aureus* contaminated topical ointments and/or specific household conditions promoting *S. aureus* colonization (i.e., less frequent handwashing, shared towel use and shared bedrooms).^39^, ^40^, ^41^, ^42^ Also, since we only used one set of confounders mostly measured at early age, residual confounding could be greater for associations between exposures and outcomes at later ages.

## 5 CONCLUSION

We observed that early life nasal carriage with *S. aureus*, but not nasopharyngeal bacterial carriage with *H. influenzae*, *M. catarrhalis* or *S. pneumoniae*, was associated with increased risks of ever eczema, and early transient and persistent eczema phenotypes. The association between nasal carriage of *S. aureus* and eczema was more prominently cross‐sectional and not longitudinal. This suggests that nasal carriage of *S. aureus* is mainly associated with active eczema, and not with eczema development in later life. Future studies should focus on the longitudinal effects of the interaction of nasal and skin microbiome within individuals and their surroundings on eczema phenotypes.

## CONFLICT OF INTEREST

The authors have no potential conflicts of interest to disclose.

## AUTHORS CONTRIBUTION

CH, LD, EM and TN contributed to the conception and design, acquisition of data, analyses and interpretation of the data, drafted the article, revised it critically for important intellectual content, and gave final approval of the version to be published. KL, JK, LP, DH, SP, JJ and HM contributed to the conception and design, acquisition of data, revised the drafted manuscript critically for important intellectual content and gave final approval of the version to be published.

## Supporting information

Supplementary Material

## Data Availability

Data requests can be made to the secretariat of the Generation R Study.

## References

[1] Deckers IA , McLean S , Linssen S , Mommers M , van Schayck CP , Sheikh A . Investigating international time trends in the incidence and prevalence of atopic eczema 1990–2010: a systematic review of epidemiological studies. PLoS One. 2012;7(7):e39803. 22808063 10.1371/journal.pone.0039803 PMC3394782

[2] Hu C , Duijts L , Erler NS , et al. Most associations of early‐life environmental exposures and genetic risk factors poorly differentiate between eczema phenotypes: the Generation R Study. Br J Dermatol. 2019;181(6):1190‐1197. 30869802 10.1111/bjd.17879 PMC6916296

[3] Paternoster L , Standl M , Waage J , et al. Multi‐ancestry genome‐wide association study of 21,000 cases and 95,000 controls identifies new risk loci for atopic dermatitis. Nat Genet. 2015;47(12):1449‐1456. 26482879 10.1038/ng.3424 PMC4753676

[4] Totté JEE , van der Feltz WT , Hennekam M , van Belkum A , van Zuuren EJ , Pasmans SGMA . Prevalence and odds of *Staphylococcus* aureus carriage in atopic dermatitis: a systematic review and meta‐analysis. Br J Dermatol. 2016;175(4):687‐695. 26994362 10.1111/bjd.14566

[5] Totte JEE , Pardo LM , Fieten KB , et al. Nasal and skin microbiomes are associated with disease severity in paediatric atopic dermatitis. Br J Dermatol. 2019;181(4):796‐804. 30737999 10.1111/bjd.17755

[6] Kumpitsch C , Koskinen K , Schöpf V , Moissl‐Eichinger C . The microbiome of the upper respiratory tract in health and disease. BMC Biol. 2019;17(1):87. 31699101 10.1186/s12915-019-0703-z PMC6836414

[7] Lebon A , Labout JA , Verbrugh HA , et al. Role of *Staphylococcus* aureus nasal colonization in atopic dermatitis in infants: the Generation R Study. Arch Pediatr Adolesc Med. 2009;163(8):745‐749. 19652107 10.1001/archpediatrics.2009.117

[8] Wang IJ , Huang LM , Guo YL , Hsieh WS , Lin TJ , Chen PC . Haemophilus influenzae type b combination vaccines and atopic disorders: a prospective cohort study. J Formos Med Assoc. 2012;111(12):711‐718. 23265751 10.1016/j.jfma.2011.09.022

[9] Arkwright PD , Patel L , Moran A , Haeney MR , Ewing CI , David TJ . Atopic eczema is associated with delayed maturation of the antibody response to pneumococcal vaccine. Clin Exp Immunol. 2000;122(1):16‐19. 11012612 10.1046/j.1365-2249.2000.01338.x PMC1905748

[10] George SMC , Karanovic S , Harrison DA , et al. Interventions to reduce *Staphylococcus* aureus in the management of eczema. Cochrane Database Syst Rev. 2019;10:CD003871. 10.1002/14651858.CD003871.pub3 PMC6818407 31684694

[11] Jaddoe VW , van Duijn CM , Franco OH , et al. The Generation R Study: design and cohort update 2012. Eur J Epidemiol. 2012;27(9):739‐756. 23086283 10.1007/s10654-012-9735-1

[12] Jaddoe VW , Mackenbach JP , Moll HA , et al. The Generation R Study: design and cohort profile. Eur J Epidemiol. 2006;21(6):475‐484. 16826450 10.1007/s10654-006-9022-0

[13] Lebon A , Labout JA , Verbrugh HA , et al. Dynamics and determinants of *Staphylococcus* aureus carriage in infancy: the Generation R Study. J Clin Microbiol. 2008;46(10):3517‐3521. 18667593 10.1128/JCM.00641-08 PMC2566071

[14] Labout JA , Duijts L , Lebon A , et al. Risk factors for otitis media in children with special emphasis on the role of colonization with bacterial airway pathogens: the Generation R study. Eur J Epidemiol. 2011;26(1):61‐66. 20821039 10.1007/s10654-010-9500-2 PMC3018595

[15] Derogatis LR , Melisaratos N . The Brief Symptom Inventory: an introductory report. Psychol Med. 1983;13(3):595‐605. 6622612

[16] van Meel ER , Jaddoe VWV , Looman KIM , de Jongste JC , Moll HA , Duijts L . Airway bacterial carriage and childhood respiratory health: a population‐based prospective cohort study. Pediatr Allergy Immunol. 2020;31(7):774‐782. 32524657 10.1111/pai.13310 PMC7587008

[17] Hu C , Nijsten T , Pasmans S , de Jongste JC , Jansen PW , Duijts L . Associations of eczema phenotypes with emotional and behavioural problems from birth until school age. The Generation R Study. Br J Dermatol. 2020;183(2):311‐320. 31730242 10.1111/bjd.18705 PMC7496612

[18] Elbert NJ , Duijts L , den Dekker HT , et al. Maternal psychiatric symptoms during pregnancy and risk of childhood atopic diseases. Clin Exp Allergy. 2017;47(4):509‐519. 28109169 10.1111/cea.12889

[19] Li MX , Yeung JM , Cherny SS , Sham PC . Evaluating the effective numbers of independent tests and significant p‐value thresholds in commercial genotyping arrays and public imputation reference datasets. Hum Genet. 2012;131(5):747‐756. 22143225 10.1007/s00439-011-1118-2 PMC3325408

[20] van Buuren S , Groothuis‐Oudshoorn K . mice: Multivariate imputation by chained equations in R. J Stat Softw. 2011;45(3):1‐67.

[21] Team RC . R: A Language and Environment for Statistical Computing. Vienna, Austria: R Foundation for Statistical Computing; 2018.

[22] Muthén LK , Muthén BO . Mplus User's Guide. 8th ed. Los Angeles, CA: Muthén & Muthén; 1998–2017.

[23] Hallquist MN , Wiley JF . MplusAutomation: An R Package for facilitating large‐scale latent variable analyses in Mplus. Struct Equ Model. 2018;25:621‐638. 10.1080/10705511.2017.1402334 PMC6075832 30083048

[24] Venables WN , Ripley BD . Modern Applied Statistics with S. 4th ed. New York, NY: Springer; 2002.

[25] Halekoh U , Højsgaard S , Yan J . The R Package geepack for generalized estimating equations. J Stat Softw. 2006;15(2):1‐11.

[26] Berbegal L , Sanchez‐Payá J , Rodriguez J , et al. Nasal colonization by *staphylococcus* aureus in children with atopic dermatitis. J Clin Exp Dermatol Res. 2018;9:4.

[27] Meylan P , Lang C , Mermoud S , et al. Skin colonization by *Staphylococcus* aureus precedes the clinical diagnosis of atopic dermatitis in infancy. J Inves Dermatol. 2017;137(12):2497‐2504. 10.1016/j.jid.2017.07.834 28842320

[28] Kennedy EA , Connolly J , Hourihane JOB , et al. Skin microbiome before development of atopic dermatitis: early colonization with commensal staphylococci at 2 months is associated with a lower risk of atopic dermatitis at 1 year. J Allergy Clin Immunol. 2017;139(1):166‐172. 27609659 10.1016/j.jaci.2016.07.029 PMC5207796

[29] Bisgaard H , Hermansen MN , Buchvald F , et al. Childhood asthma after bacterial colonization of the airway in neonates. N Engl J Med. 2007;357(15):1487‐1495. 17928596 10.1056/NEJMoa052632

[30] Jung JA , Kita H , Yawn BP , et al. Increased risk of serious pneumococcal disease in patients with atopic conditions other than asthma. J Allergy Clin Immunol. 2010;125(1):217‐221. 20109748 10.1016/j.jaci.2009.10.045 PMC2825162

[31] Geoghegan JA , Irvine AD , Foster TJ . *Staphylococcus* aureus and atopic dermatitis: a complex and evolving relationship. Trends Microbiol. 2018;26(6):484‐497. 29233606 10.1016/j.tim.2017.11.008

[32] Hong SW , Kim MR , Lee EY , et al. Extracellular vesicles derived from *Staphylococcus* aureus induce atopic dermatitis‐like skin inflammation. Allergy. 2011;66(3):351‐359. 20831718 10.1111/j.1398-9995.2010.02483.x PMC3052535

[33] Hepburn L , Hijnen DJ , Sellman BR , et al. The complex biology and contribution of *Staphylococcus* aureus in atopic dermatitis, current and future therapies. Br J Dermatol. 2017;177(1):63‐71. 27779765 10.1111/bjd.15139

[34] Zhang X , Zhivaki D , Lo‐Man R . Unique aspects of the perinatal immune system. Nat Rev Immunol. 2017;17(8):495‐507. 28627520 10.1038/nri.2017.54

[35] Feuillie C , Vitry P , McAleer MA , et al. Adhesion of *Staphylococcus* aureus to corneocytes from atopic dermatitis patients is controlled by natural moisturizing factor levels. mBio. 2018;9(4):e01184‐18. 30108169 10.1128/mBio.01184-18 PMC6094479

[36] Sakr A , Brégeon F , Mège J‐L , Rolain J‐M , Blin O . *Staphylococcus* aureus nasal colonization: an update on mechanisms, epidemiology, risk factors, and subsequent infections. Front Microbiol. 2018;9:2419. 30349525 10.3389/fmicb.2018.02419 PMC6186810

[37] Turner NA , Sharma‐Kuinkel BK , Maskarinec SA , et al. Methicillin‐resistant *Staphylococcus* aureus: an overview of basic and clinical research. Nat Rev Microbiol. 2019;17(4):203‐218. 30737488 10.1038/s41579-018-0147-4 PMC6939889

[38] Silverberg JI , Patel N , Immaneni S , et al. Assessment of atopic dermatitis using self‐report and caregiver report: a multicentre validation study. Br J Dermatol. 2015;173(6):1400‐1404. 26186170 10.1111/bjd.14031 PMC5216166

[39] Gilani SJ , Gonzalez M , Hussain I , Finlay AY , Patel GK . *Staphylococcus* aureus re‐colonization in atopic dermatitis: beyond the skin. Clin Exp Dermatol. 2005;30(1):10‐13. 15663492 10.1111/j.1365-2230.2004.01679.x

[40] Mork RL , Hogan PG , Muenks CE , et al. Longitudinal, strain‐specific *Staphylococcus* aureus introduction and transmission events in households of children with community‐associated meticillin‐resistant *S aureus* skin and soft tissue infection: a prospective cohort study. Lancet Infect Dis. 2020;20(2):188‐198. 31784369 10.1016/S1473-3099(19)30570-5 PMC6995751

[41] Chiu LS , Chow VC , Ling JM , Hon KL . *Staphylococcus* aureus carriage in the anterior nares of close contacts of patients with atopic dermatitis. Arch Dermatol. 2010;146(7):748‐752. 20644035 10.1001/archdermatol.2010.129

[42] Bonness S , Szekat C , Novak N , Bierbaum G . Pulsed‐field gel electrophoresis of *staphylococcus* aureus isolates from atopic patients revealing presence of similar strains in isolates from children and their parents. J Clin Microbiol. 2008;46(2):456‐461. 18077648 10.1128/JCM.01734-07 PMC2238135

