# Supplementary material for: Association between nasal and nasopharyngeal bacterial colonization in early life and eczema phenotypes
Source: Clin Exp Allergy. 2021 Apr 6;51(5):716–25. doi: 10.1111/cea.13869 (PMC8252109; doi:10.1111/cea.13869)

**Supplemental materials**

**Association between nasal and nasopharyngeal bacterial colonisation in early life and eczema phenotypes.**

Chen Hu^1,2^, Liesbeth Duijts^3,4^, Evelien R. van Meel^1,3^, Kirsten I.M. Looman^1,5^, Jessica C. Kiefte-de Jong^6,7^, Luba M. Pardo^2^, DirkJan Hijnen^2^, Suzanne G.M.A. Pasmans^2^, Johan C. de Jongste^3^, Henriette A. Moll^5^, Tamar Nijsten^2^

^1^The Generation R Study Group, Erasmus MC, University Medical Center Rotterdam, Rotterdam, The Netherlands. ^2^Department of Dermatology, Erasmus MC, University Medical Center Rotterdam, Rotterdam, The Netherlands. ^3^Department of Pediatrics, Division of Respiratory Medicine and Allergology, Erasmus MC, University Medical Center Rotterdam, Rotterdam, The Netherlands. ^4^Department of Pediatrics, division of Neonatology, Erasmus MC, University Medical Center Rotterdam, Rotterdam, The Netherlands. ^5^Department of Pediatrics, Erasmus MC, University Medical Center Rotterdam, Rotterdam, The Netherlands. ^6^Department of Epidemiology, Erasmus MC, University Medical Center Rotterdam, Rotterdam, The Netherlands. ^7^Department of Public Health and Primary Care, Leiden University Medical Center, Leiden, The Netherlands

**Corresponding author:**

Tamar Nijsten, Department of Dermatology, Erasmus Medical Center Rotterdam, PO Box 2040, 3000 CB Rotterdam, The Netherlands.

Tel: +31 10 7034580 , E-mail: t.nijsten@erasmusmc.nl

**Table S1. Characteristics of children included and not included**

|  | **Included**  (n=996) | **Not included**  (n=250) | **p-value** |
| --- | --- | --- | --- |
| **Maternal characteristics** |  |  |  |
| Pet keeping % (n) |  |  | 0.140 |
| No | 57.6 (510) | 51.9 (120) |  |
| Yes | 42.4 (375) | 48.1 (111) |  |
| Pyschiatric symptoms, median (IQR) | 0.12 (0.06, 0.23) | 0.13 (0.06, 0.29) | 0.017 |
| Mode of delivery % (n) |  |  | 0.435 |
| Vaginal | 85.6 (786) | 87.3 (186) |  |
| Primary caesarian section | 6.0 (55) | 3.8 (8) |  |
| Secondary caesarian section | 8.4 (77) | 8 .9 (19) |  |
| **Children’s characteristics** |  |  |  |
| Day care attendance 1^st^ year % (n) |  |  | 0.194 |
| No | 30.8 (272) | 37.8 (37) |  |
| Yes | 69.2 (612) | 62.2 (61) |  |
| Antibiotic use at age 1 year % (n) |  |  | 0.395 |
| No | 64.3 (603) | 59.6 (65) |  |
| Yes | 35.7 (335) | 40.4 (44) |  |
| Eczema % (n) |  |  |  |
| Age 6 months, No | 86.6 (587) | 88.4 (76) | 0.769 |
| Yes | 13.4 (91) | 11.6 (10) |  |
| Age 1 year, No | 88.4 (823) | 89.8 (97) | 0.781 |
| Yes | 11.6 (108) | 10.2 (11) |  |
| Age 2 years, No | 87.0 (815) | 85.7 (78) | 0.858 |
| Yes | 13.0 (122) | 14.3 (13) |  |
| Age 3 years, No | 93.0 (837) | 95.1 (58) | 0.718 |
| Yes | 7.0 (63) | 4.9 (3) |  |
| Age 4 years, No | 93.5 (829) | 93.7 (59) | 1.000 |
| Yes | 6.5 (58) | 6.3 (4) |  |
| Age 10 years, No | 94 (778) | 95.6 (43) | 0.907 |
| Yes | 6.0 (50) | 4.4 (2) |  |

Values are percentages (absolute values), mean (SD) or median (interquartile range) based on observed data.

**Table S2. Associations of bacterial nasal carriage with regrouped three eczema phenotypes**

|  | **Never eczema**  **Odds ratio (95% Confidence Interval)**  (n=768) | **Early-mid transient eczema**  **Odds ratio (95% Confidence Interval)**  (n=142) | **Late-persistent eczema**  **Odds ratio (95% Confidence Interval)**  (n=86) |
| --- | --- | --- | --- |
| ***S. aureus* carriage** |  |  |  |
| Age 6 weeks | Reference | 1.52 (0.82, 2.81) | 0.90 (0.48, 1.69) |
| Age 6 months | Reference | 2.39 (1.37, 4.15)** | 1.88 (0.98, 3.61) |
| Age 1 year | Reference | 0.98 (0.47, 2.06) | 0.86 (0.35, 2.09) |
| Age 2 years | Reference | 1.74 (0.87, 3.49) | 1.36 (0.55, 3.33) |
| Age 3 years | Reference | 1.68 (0.83, 3.40) | 1.31 (0.58, 2.97) |
| Age 6 years | Reference | 1.28 (0.77, 2.14) | 1.73 (0.99, 3.00) |
| **Nasopharyngeal carriage with any bacteria**^†^ | | | |
| Age 6 weeks | Reference | 1.22 (0.60, 2.46) | 1.76 (0.89, 3.50) |
| Age 6 months | Reference | 1.13 (0.65, 1.97) | 0.95 (0.51, 1.76) |
| Age 1 year | Reference | 0.94 (0.52, 1.72) | 0.92 (0.46, 1.84) |
| Age 2 years | Reference | 0.89 (0.51, 1.57) | 1.12 (0.55, 2.26) |
| Age 3 years | Reference | 1.03 (0.59, 1.80) | 0.95 (0.52, 1.74) |
| Age 6 years | Reference | 1.06 (0.66, 1.72) | 1.14 (0.67, 1.94) |

Values are odds ratios (OR) with 95% confidence interval from multinomial regression models on imputed data. ^†^Nasopharyngeal bacteria include *H. influenzae, M. catarrhalis* or *S. pneumoniae.* Models were adjusted for maternal psychiatric symptoms, pet keeping, mode of delivery, daycare attendance and antibiotic use. *p-value <0.05, **p-value <0.01.

**Table S3. Associations of nasopharyngeal carriage of *H. influenzae, M. catarrhalis* or *S. pneumoniae* with ever eczema and eczema phenotypes**

|  | **Ever eczema**  **Odds ratio (95% Confidence Interval)**  (n=228) | **Never eczema**  **Odds ratio (95% Confidence Interval)**  (n=768) | **Early transient eczema**  **Odds ratio (95% Confidence Interval)**  (n=75) | **Mid-transient eczema**  **Odds ratio (95% Confidence Interval)**  (n=67) | **Late transient eczema**  **Odds ratio (95% Confidence Interval)**  (n=71) | **Persistent eczema**  **Odds ratio (95% Confidence Interval)**  (n=15) | |
| --- | --- | --- | --- | --- | --- | --- | --- |
| ***H. influenzae* carriage** | | | | | | | |
| Age 6 weeks | 1.07 (0.46, 2.45) | Reference | 0.43 (0.05, 3.60) | 2.61 (0.56, 12.22) | 1.20 (0.33, 4.39) | 0.00 (0.00, >100) | |
| Age 6 months | 1.27 (0.84, 1.93) | Reference | 2.09 (1.03, 4.24) | 1.21 (0.46, 3.20) | 0.53 (0.21, 1.38) | 1.96 (0.50, 7.65) | |
| Age 1 year | 0.78 (0.51, 1.19) | Reference | 1.32 (0.65, 2.67) | 0.30 (0.08, 1.09) | 0.86 (0.40, 1.87) | 0.51 (0.09, 3.00) | |
| Age 2 years | 1.02 (0.64, 1.62) | Reference | 0.88 (0.40, 1.95) | 1.09 (0.38, 3.11) | 1.15 (0.49, 2.67) | 0.61 (0.06, 6.04) | |
| Age 3 years | 0.99 (0.62, 1.60) | Reference | 1.03 (0.42, 2.53) | 0.93 (0.30, 2.87) | 1.12 (0.51, 2.50) | 1.41 (0.22, 8.88) | |
| Age 6 years | 0.89 (0.52, 1.51) | Reference | 0.89 (0.32, 2.48) | 0.85 (0.25, 2.92) | 0.93 (0.36, 2.42) | 1.62 (0.29, 8.89) | |
| ***M. catarrhalis* carriage** | | | | | | | |
| Age 6 weeks | 1.28 (0.68, 2.43) | Reference | 1.23 (0.36, 4.21) | 0.53 (0.07, 4.04) | 1.96 (0.78, 4.92) | 1.54 (0.15, 15.84) | |
| Age 6 months | 0.99 (1.67, 1.72) | Reference | 0.89 (0.43, 1.84) | 1.33 (0.54, 3.29) | 0.99 (0.48, 2.06) | 0.66 (0.14, 3.06) | |
| Age 1 year | 0.92 (0.61, 1.40) | Reference | 1.08 (0.52, 2.24) | 0.91 (0.33, 2.48) | 0.75 (0.34, 1.67) | 1.05 (0.25, 4.43) | |
| Age 2 years | 0.88 (0.17, 0.46) | Reference | 0.79 (0.37, 1.69) | 1.16 (0.43, 3.11) | 0.90 (0.40, 2.03) | 0.17 (0.01, 2.35) | |
| Age 3 years | 0.94 (0.59, 1.50) | Reference | 1.82 (0.82, 4.03) | 0.82 (0.27, 2.54) | 1.04 (0.47, 2.27) | 0.00 (0.00, >100) | |
| Age 6 years | 0.81 (0.46, 1.41) | Reference | 0.74 (0.25, 2.19) | 0.87 (0.24, 3.18) | 0.82 (0.30, 2.23) | 0.62 (0.06, 5.97) | |
| ***S. pneumonia*e carriage** | | | | | | |  |
| Age 6 weeks | 1.13 (0.55, 2.31) | Reference | 0.78 (0.16, 3.67) | 1.03 (0.19, 5.65) | 1.14 (0.35, 3.73) | 1.58 (0.13, 19.21) | |
| Age 6 months | 0.92 (0.62, 1.38) | Reference | 0.92 (0.44, 1.91) | 1.12 (0.45, 2.81) | 0.76 (0.35, 1.64) | 0.73 (0.15, 3.64) | |
| Age 1 year | 0.97 (0.65, 1.43) | Reference | 1.05 (0.52, 2.09) | 0.84 (0.33, 2.17) | 0.98 (0.47, 2.02) | 0.75 (0.18, 3.19) | |
| Age 2 years | 0.78 (0.51, 1.19) | Reference | 0.51 (0.24, 1.09) | 1.13 (0.44, 2.91) | 0.61 (0.27, 1.38) | 1.30 (0.27, 6.26) | |
| Age 3 years | 0.79 (0.51, 1.23) | Reference | 1.11 (0.50, 2.46) | 0.41 (0.12, 1.46) | 1.02 (0.49, 2.09) | 1.18 (0.22, 6.28) | |
| Age 6 years | 1.07 (0.73, 1.57) | Reference | 0.92 (0.43, 1.94) | 1.29 (0.57, 2.95) | 1.02 (0.52, 2.03) | 1.17 (0.28, 4.82) | |

Values are odds ratios (OR) with 95% confidence interval from logistic and multinomial regression models on imputed data. Models were adjusted for maternal psychiatric symptoms, pet keeping, mode of delivery, daycare attendance and antibiotic use. *adjusted p-value < 0.05

**Table S4.** Direction of associations between bacterial nasal and nasopharyngeal carriage^†^ and eczema from birth until age 10 years

|  | ***S. Aureus* carriage** | **Nasopharyngeal carriage with any bacteria** |
| --- | --- | --- |
| **Cross-lagged effects** |  |  |
| Bacterial nasal carriage 6w → Eczema 6m | 1.09 (0.64, 1.86) | 1.28 (0.68, 2.46) |
| Bacterial nasal carriage 6m → Eczema 1y | 1.35 (0.76, 2.44) | 1.00 (0.58, 1.72) |
| Bacterial nasal carriage 1y → Eczema 2y | 1.11 (0.61, 2.01) | 0.79 (0.47, 1.32) |
| Bacterial nasal carriage 2y → Eczema 3y | 0.85 (0.35, 2.12) | 0.90 (0.47, 1.75) |
| Bacterial nasal carriage 3y → Eczema 4y | 1.28 (0.48, 3.42) | 1.73 (0.85, 3.53) |
| Bacterial nasal carriage 6y → Eczema 10y | 1.35 (0.70, 2.61) | 1.19 (0.64, 2.20) |
| Eczema 6m → Bacterial nasal carriage 1y | 0.52 (0.18, 1.46) | 0.82 (0.43, 1.57) |
| Eczema 1y → Bacterial nasal carriage 2y | 1.05 (0.52, 2.10) | 0.75 (0.44, 1.28) |
| Eczema 2y → Bacterial nasal carriage 3y | 1.95 (1.02, 3.71)* | 1.09 (0.64, 1.86) |
| Eczema 4y → Bacterial nasal carriage 6y | 1.46 (0.79, 2.72) | 1.22 (0.68, 2.20) |
| **Cross-sectional effects** |  |  |
| Bacterial nasal carriage ↔ Eczema 6m | 2.39 (1.38, 4.14)** | 1.02 (0.61, 1.73) |
| Bacterial nasal carriage ↔ Eczema 1y | 1.60 (0.76, 3.39) | 1.05 (0.57, 1.93) |
| Bacterial nasal carriage ↔ Eczema 2y | 2.20 (1.16, 4.18)* | 1.09 (0.63, 1.90) |
| Bacterial nasal carriage ↔ Eczema 3y | 0.85(0.36, 2.01) | 0.65 (0.34, 1.25) |
| **Stability effects** |  |  |
| Bacterial nasal carriage 6w → 6m | 2.23 (1.36, 3.60)** | 1.72 (1.05, 2.83)* |
| Bacterial nasal carriage 6m → 1y | 1.42 (0.82, 2.44) | 3.32 (2.29, 4.81)** |
| Bacterial nasal carriage 1y → 2y | 0.92 (0.41, 2.08) | 2.29 (1.54, 3.39)** |
| Bacterial nasal carriage 2y → 3y | 2.39 (1.27, 4.48)** | 1.28 (0.86, 1.92) |
| Bacterial nasal carriage 3y → 6y | 1.73 (1.04, 2.89)* | 1.63 (1.15, 2.34)** |
| Eczema 6m → 1y | 10.07 (5.75, 17.64)** | 11.02 (6.36, 18.92)** |
| Eczema 1y → 2y | 8.50 (5.31, 13.46)** | 8.67 (5.42, 13.74)** |
| Eczema 2y → 3y | 6.49 (3.71, 11.36)** | 6.42 (3.71, 11.13)** |
| Eczema 3y → 4y | 7.54 (3.82, 14.88)** | 8.17 (4.06, 16.28)** |
| Eczema 4y → 10y | 4.53 (1.99, 10.28)** | 4.66 (2.12, 10.38)** |

Values are odds ratios (95% confidence interval) derived from logistic regression models, using cross-lagged modelling. ^†^Nasopharyngeal bacteria include *H. influenzae, M. catarrhalis* or *S. pneumoniae.* Models were adjusted for maternal psychiatric symptoms, pet keeping, mode of delivery, daycare attendance and antibiotic use. *p-value <0.05, **p-value <0.01. Abbreviation used: weeks (w), months (m), years (y).

**Table S5.** Direction of associations between bacterial nasopharyngeal carriage and eczema from birth until age 10 years

|  | ***H. influenzae* carriage** | ***M. catarrhalis*** **carriage** | ***S. pneumonia*** **carriage** |
| --- | --- | --- | --- |
| **Cross-lagged effects** |  |  |  |
| Bacterial nasal carriage 6w → Eczema 6m | 0.84 (0.28, 2.51) | 1.35 (0.55, 3.32) | 1.23 (0.47, 3.22) |
| Bacterial nasal carriage 6m → Eczema 1y | 1.80 (1.03, 3.13) | 1.19 (0.68, 2.10) | 0.59 (0.33, 1.05) |
| Bacterial nasal carriage 1y → Eczema 2y | 0.53 (0.28, 0.99) | 0.70 (0.39, 1.25) | 1.01 (0.61, 1.68) |
| Bacterial nasal carriage 2y → Eczema 3y | 1.17 (0.57, 2.44) | 0.68 (0.31, 1.46) | 0.45 (0.21, 0.96) |
| Bacterial nasal carriage 3y → Eczema 4y | 1.79 (0.84, 3.82) | 1.49 (0.69, 3.19) | 1.46 (0.72, 2.94) |
| Bacterial nasal carriage 6y → Eczema 10y | 1.75 (0.77, 3.97) | 1.02 (0.38, 2.75) | 0.90 (0.44, 1.88) |
| Eczema 6m → Bacterial nasal carriage 1y | 0.83 (0.44, 1.54) | 0.99 (0.53, 1.86) | 0.79 (0.43, 1.46) |
| Eczema 1y → Bacterial nasal carriage 2y | 0.97 (0.52, 1.80) | 0.66 (0.37, 1.20) | 0.70 (0.39, 1.22) |
| Eczema 2y → Bacterial nasal carriage 3y | 0.99 (0.50, 1.99) | 0.99 (0.51, 1.93) | 0.76 (0.41, 1.42) |
| Eczema 4y → Bacterial nasal carriage 6y | 1.40 (0.61, 3.29) | 1.21 (0.5, 2.92) | 0.86 (0.43, 1.73) |
| **Cross-sectional effects** |  |  |  |
| Bacterial nasal carriage ↔ Eczema 6m | 1.14 (0.64, 2.03) | 0.95 (0.53, 1.70) | 1.17 (0.69, 1.99) |
| Bacterial nasal carriage ↔ Eczema 1y | 0.85 (0.51, 1.45) | 1.14 (0.65, 1.99) | 1.35 (0.79, 2.32) |
| Bacterial nasal carriage ↔ Eczema 2y | 0.75 (0.38, 1.49) | 0.99 (0.55, 1.79) | 1.13 (0.65, 1.95) |
| Bacterial nasal carriage ↔ Eczema 3y | 0.85 (0.37, 1.95) | 0.61 (0.26, 1.48) | 1.00 (0.49, 2.05) |
| **Stability effects** |  |  |  |
| Bacterial nasal carriage 6w → 6m | 2.80 (1.23, 6.42) | 1.08 (0.55, 2.12) | 1.45 (0.74, 2.83) |
| Bacterial nasal carriage 6m → 1y | 2.01 (1.32, 3.06)* | 1.62 (1.11, 2.39) | 2.97 (2.05, 4.35)* |
| Bacterial nasal carriage 1y → 2y | 1.82 (1.17, 2.80)* | 1.20 (0.78, 1.84) | 1.65 (1.13, 2.41)* |
| Bacterial nasal carriage 2y → 3y | 1.45 (0.86, 2.46) | 0.85 (0.51, 1.43) | 1.92 (1.25, 2.97)* |
| Bacterial nasal carriage 3y → 6y | 0.87 (0.42, 1.79) | 1.60 (0.88, 2.92) | 1.62 (1.06, 2.46) |
| Eczema 6m → 1y | 11.13 (6.42, 19.3)* | 10.91 (6.36, 18.92)* | 11.36 (6.55, 19.69)* |
| Eczema 1y → 2y | 8.58 (5.37, 13.74)* | 8.76 (5.47, 13.87)* | 8.58 (5.42, 13.60)* |
| Eczema 2y → 3y | 6.55 (3.78, 11.36)* | 6.42 (3.71, 11.13)* | 6.62 (3.78, 11.59)* |
| Eczema 3y → 4y | 7.69 (3.94, 15.18)* | 8.00 (4.06, 15.80)* | 7.85 (3.97, 15.49)* |
| Eczema 4y → 10y | 4.71 (2.12, 10.49)* | 4.62 (2.08, 10.28)* | 4.76 (2.16, 10.49)* |

Values are odds ratios (95% confidence interval) derived from logistic regression models, using cross-lagged modelling. Models were adjusted for maternal psychiatric symptoms, pet keeping, mode of delivery, daycare attendance and antibiotic use. *adjusted p-value <0.05. Abbreviation used: weeks (w), months (m), years (y).

**Figure S1**. Overview of cross-lagged model design


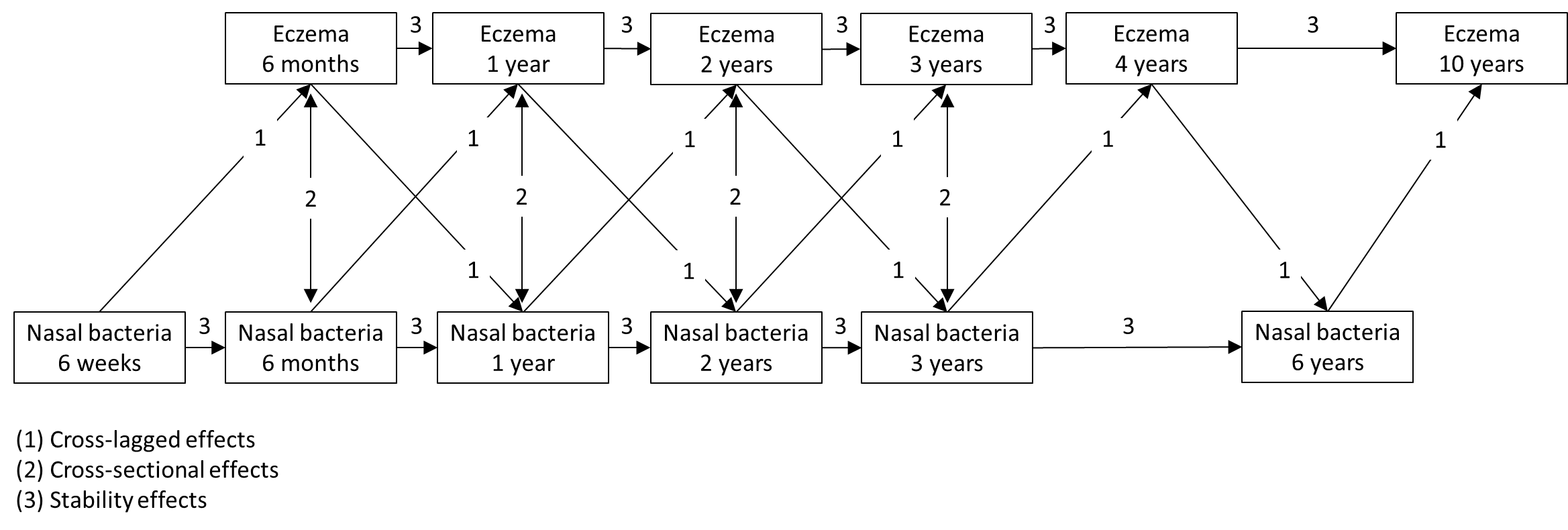

Supplement: Supplementary file 1 — Supplementary Material [file CEA-51-716-s001.doc]
